# Associations between vertebral bone marrow fat and sagittal spine alignment as assessed by chemical shift-encoding-based water–fat MRI

**DOI:** 10.1186/s13018-023-03944-w

**Published:** 2023-06-27

**Authors:** Fangsi Chen, Yingying Huang, Anna Guo, Peipei Ye, Jiawei He, Shaoqing Chen

**Affiliations:** 1 Department of Radiology, The Second Affiliated Hospital and Yuying Children’s Hospital of Wenzhou Medical University, 109 Xueyuanxi Rd, Wenzhou, 325027 Zhejiang China; 2 Department of Radiology, Wenzhou Seventh People’s Hospital, Wenzhou, China

**Keywords:** Bone marrow fat, Sagittal spine alignment, IDEAL IQ, Fat fraction, Spinal degeneration

## Abstract

**Background:**

The relationship between sagittal spine alignment and vertebral bone marrow fat is unknown. We aimed to assess the relationship between vertebral bone marrow fat and sagittal spine alignment using chemical shift-encoding-based water–fat magnetic resonance imaging (MRI).

**Methods:**

A total of 181 asymptomatic volunteers were recruited for whole spine X-ray and lumbar MRI. Spine typing was performed according to the Roussouly classification and measurement of vertebral fat fraction based on the chemical shift-encoding-based water–fat MRI. One-way analysis of variance (ANOVA) was used to analyze the differences in vertebral fat fraction between spine types. The post hoc least significant difference (LSD) test was utilized for subgroup comparison after ANOVA.

**Results:**

Overall, the vertebral fat fraction increased from L1 to L5 and was the same for each spine type. The vertebral fat fraction was the highest in type 1 and lowest in type 4 at all levels. ANOVA revealed statistically significant differences in fat fraction among different spine types at L4 and L5 (*P* < .05). The post hoc LSD test showed that the fat fraction of L4 was significantly different (*P* < .05) between type 1 and type 4 as well as between type 2 and type 4. The fat fraction of L5 was significantly different between type 1 and type 3, between type 1 and type 4, and between type 2 and type 4 (*P* < .05).

**Conclusion:**

Our study found that vertebral bone marrow fat is associated with sagittal spine alignment, which may serve as a new additional explanation for the association of sagittal alignment with spinal degeneration.

## Background

Bone marrow, which consists of yellow and red marrow, serves as important hematopoietic and immune tissues, with adipose tissue being a key component of bone marrow. Over time, research has confirmed that bone marrow adipocytes and osteoblasts originate from a common precursor—bone marrow mesenchymal stem cells—and that an increase in adipocytes will be accompanied by a decrease in osteoblasts [1–3]. The conversion of the hematopoietic bone marrow to bone marrow fat (BMF) accompanies a decrease in vertebral perfusion and nutrition supplies, which demonstrates that adipose tissue is an important factor affecting the bone marrow microenvironment [4].

Chemical shift-encoding-based water–fat MRI is a highly efficient technique for determining fat and water signals in humans, making it possible to quantify water–fat composition noninvasively [5, 6]. The iterative decomposition of water and fat with echo asymmetry and least squares estimation (IDEAL IQ) was developed from the Dixon technique, which corrects for T2* attenuation and fat multispectral peak distribution using an asymmetric acquisition technique and an iterative least squares water–fat separation algorithm, converting the water–fat separation from qualitative to quantitative. Fat, fat fraction, water and R2* relaxation images can be generated in a single scan, and the BMF can be quantified from fat fraction images [2, 3, 7, 8]. IDEAL IQ can adjust for common biases in tissue fat measurements, including T1 bias and T2* effects and main magnetic field inhomogeneities. Therefore, it has been confirmed as one of the most convenient and accurate techniques to quantify BMF.

The spine is the most important support structure in the human body, and there is a consensus that intervertebral disk (IVD) degeneration is closely related to the biomechanics of sagittal spine alignment [9–11]. The IVD is the largest avascular tissue in the body, and its health depends on nutrient transport from the capillaries of the adjacent vertebral body, while the conversion of hematopoietic bone marrow to fatty bone marrow is accompanied by a decrease in vertebral perfusion and nutrient supply. It has also been suggested that inflammatory components released by the IVD may trigger an autoimmune response that leads to bone marrow damage in the adjacent vertebrae, thereby accelerating this process [12, 13]. In line with these theories, a recent study by Krug et al. [14] found a correlation between early IVD degeneration and vertebral body fat content. As the most important weight-bearing part of the spine, the lumbar spine is also the most prone to degeneration. Many studies have analyzed the relationship between lumbar IVD degeneration and sagittal spine alignment. To date, however, no study has evaluated the correlation between sagittal spine alignment and vertebral BMF.

The purpose of this study was to assess the relationship between vertebral BMF and sagittal spine alignment using the IDEAL IQ.

## Methods

### Subjects

We recruited 181 asymptomatic volunteers (75 men, 106 women, average age 35.25 ± 8.57 years) from 2016 to 2021. The study was approved by the local ethics committee review board. The purpose of the study was communicated to the volunteers, and they provided written informed consent before participating in the study. The inclusion criteria were as follows: (1) age 20 to 50 years; (2) no history of spinal surgery; (3) no arthropathy of the lower extremities; and (4) no history of neuromuscular disease. The exclusion criteria were as follows: (1) low back pain, neck pain, or numbness in the extremities due to spinal diseases; (2) occupations involving heavy physical labor; (3) spinal trauma or tumors; and (4) spinal deformities (including scoliosis, isthmus crack, and irregular endplates). Demographic characteristics of the participants were collected, including age, sex, weight, height, and body mass index (BMI).

### Full-length X-ray of the spine

**Fig. 1:**
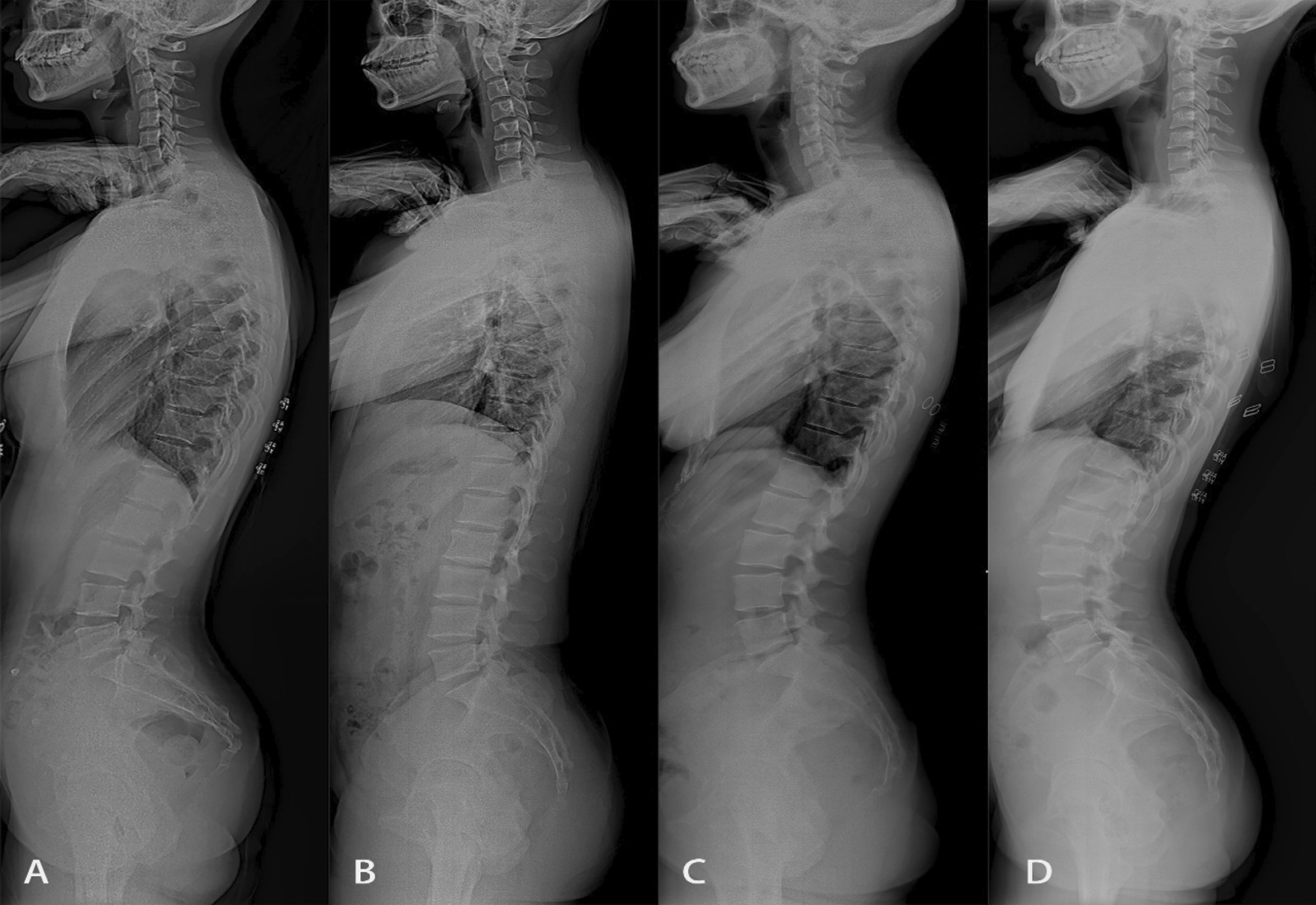
Types 1 to 4 **A**–**D** in the classic Roussouly classification

Standing lateral radiographs of the full spine were obtained using a Siemens digital radiography system (Siemens YSIO, Siemens, Germany) and Picture Archiving and Communication System (PACS) v3.0 (INFINITT, Shanghai, China). The radiographs were taken in a standard position, requiring the subject to stand naturally, with hands on the clavicle and hips and knees extended. A spine surgeon and a radiologist examined the radiographs separately. On the basis of the radiographs, the spines were divided into four types by two experienced radiologists according to the classic Roussouly classification (Fig. 1). Spine typing is a comprehensive evaluation of sagittal morphology that is more reasonable than a single sagittal parameter, and Roussouly typing is the most recognized sagittal typology of the spine based on spinal pelvic parameters [15]. Each type represents a distinct spinopelvic morphological complex. When the SS is less than 35°, the lumbar lordosis (LL) is small and the thoracic kyphosis (TK) is large, the spine is classified as type 1. When the distal arches are lower and larger, the LL will tend to flatten, and the spine is classified as type 2. When SS is between 35 and 45° and TK is coordinated with LL, the spine is classified as type 3. When the LL is greater than 45°, the lumbar curvature will be increased and the TK reduced, a spine with these features is classified as type 4. When two observers disagreed on the typing of the spine, a third observer (a radiologist with more than 20 years of experience in diagnostic musculoskeletal radiology) was included in the consultation and determination.

### Magnetic resonance examination and image processing

All lumbar spine scans were performed on a 3.0 T superconducting MR scanner (GE Discovery MR 750) using an eight-channel spine phased-array coil with the patient in the supine position. Water–fat MRI consisted of a three-dimensional spoiled gradient sequence with six echoes and IDEAL reconstruction algorithm [16]. The sequence was performed with the following scan parameters: TR/TE_1_/ΔTE = 6.4/1.0/0.8 ms, echo train length = 3, number shots = 2, bandwidth = 868 Hz/pixel, frequency direction = A/P, FOV = 35 cm, in-plane resolution = 2.1 mm, slice thickness = 10 mm, flip angle = 3°, NEX = 1, and scan time = 1 min and 7 s.

**Fig. 2:**
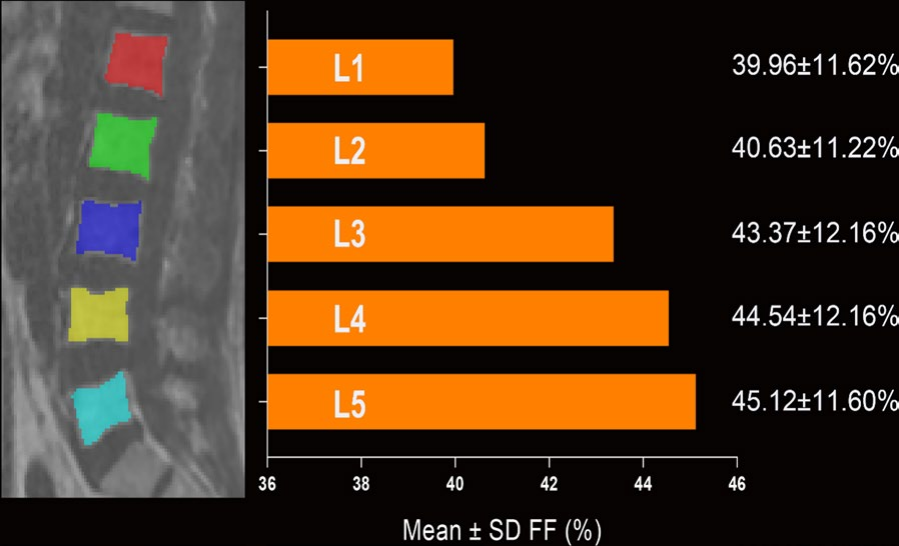
Mean and SD of FF for each lumbar level overall. A fat fraction image is shown on the left to illustrate how we outlined the ROIs at different lumbar levels. The FF by level ranged from 39% in L1 to 45% in L5

The IDEAL processing was performed on the scanner using the vendor's implementation (online). And the region of interest (ROI) was outlined independently at the sagittal level of the lumbar vertebral body by two experienced radiologists. The ROI should include as much of the cancellous bone of each vertebral body as possible, avoiding the cortical bone and the entrance to the vertebral vein as well (Fig. 2). The FF of each vertebra was recorded, and finally, the average was taken to reduce the error.

### Statistical analysis

Statistical analyses were performed using SPSS v.25.0 statistical software. The mean ± standard deviation (SD) was calculated for all applicable data. Tests of normality and homogeneity of variance were performed before analysis. One-way analysis of variance (ANOVA) and Chi-square tests were used to test whether there were differences in demographic characteristics (sex, age, and BMI) between different spine types. One-way ANOVA was used to analyze whether there was a difference in FF between vertebrae of different spine types. The post hoc least significant difference (LSD) test was utilized for subgroup comparisons after ANOVA. The statistical significance criterion was defined as *P* < .05.

## Results

**Fig. 3:**
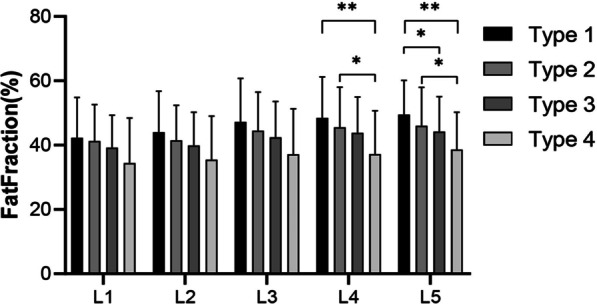
FF at each level for each of the four spine types

The data of all groups conformed to the normal distribution and the Chi-square distribution. There were no significant differences in sex, age, or BMI between the groups (*P* > .05). The FF of the lumbar spine increased from L1 to L5 in all asymptomatic volunteers (L1 39.96 ± 11.26%, L2 40.63 ± 11.22%, L3 43.37 ± 12.16%, L4 44.54 ± 12.165, L5 45.12 ± 11.60%), and all spinal types conformed to this pattern (Fig. 2 and Table 1). Of the four spine types, FF was the highest in type 1, followed by type 2 and type 3, and lowest for type 4 (Table 1). A one-way ANOVA revealed statistically significant differences in FF at L4 and L5 (L4 *P* = .037, L5 *P* = .032) (Table 1). The post hoc LSD test showed significant differences between type 1 and type 4 (type 1 48.53 ± 12.70%, type 4 37.24 ± 13.44%, *P* = .005) and between type 2 and type 4 (type 2 45.56 ± 12.45%, type 4 37.24 ± 13.44%, *P* = .021) at L4 (Table 2 and Fig. 3). There were also significant differences between type 1 and type 3 (type 1 49.50 ± 10.69%, type 3 44.26 ± 10.86%, *P* = .046), between type 1 and type 4 (type 1 49.50 ± 10.69%, type 4 38.66 ± 11.60%, *P* = .005), and between type 2 and type 4 (type 2 46.01 ± 11.98%, type 4 38.66 ± 11.60%, *P* = .032) at L5 (Table 2 and Fig. 3).

**Table 1:** Comparison of demographic parameters and FF among spine types

	Total	Type 1	Type 2	Type 3	Type 4	*P* value
*N*	181	25	59	83	14	
Sex (M/F)	75/106	11/14	25/34	37/46	2/12	.197
Age (years)	35.25 ± 8.57	36.72 ± 7.92	34.85 ± 8.91	35.13 ± 8.62	35.07 ± 8.55	.831
BMI (m/kg^2^)	22.61 ± 3.06	23.81 ± 3.24	22.21 ± 2.61	22.65 ± 3.27	21.94 ± 2.85	.136
*FF* (mean ± SD %)						
L1	39.96 ± 11.26	42.33 ± 12.47	41.30 ± 11.34	39.24 ± 10.09	34.41 ± 13.99	.127
L2	40.63 ± 11.22	44.01 ± 12.74	41.47 ± 10.90	39.87 ± 10.35	35.51 ± 13.49	.117
L3	43.37 ± 12.16	47.23 ± 13.52	44.51 ± 12.01	42.43 ± 11.15	37.17 ± 14.10	.065
L4	44.54 ± 12.16	48.53 ± 12.70	45.56 ± 12.45	43.83 ± 11.14	37.24 ± 13.44	.037*
L5	45.12 ± 11.60	49.50 ± 10.69	46.01 ± 11.98	44.26 ± 10.86	38.66 ± 11.60	.032*

**Table 2:** Comparison of the FF of L4 and L5 among spine types

	FF (mean ± SD %)				*P* value for the comparison of different spine types					
	Type 1	Type 2	Type 3	Type 4	1 vs. 2	1 vs. 3	1 vs. 4	2 vs. 3	2 vs. 4	3 vs. 4
L4	48.53 ± 12.70	45.56 ± 12.45	43.83 ± 11.14	37.24 ± 13.44	0.300	0.087	0.005*	0.399	0.021*	0.058
L5	49.50 ± 10.69	46.01 ± 11.98	44.26 ± 10.86	38.66 ± 11.60	0.201	0.046*	0.005*	0.371	0.032*	0.091

## Discussion

The IDEAL IQ can sensitively detect changes in the fat content of bone marrow and achieve wide coverage in a short scan time [17, 18]. Therefore, it may be a better choice than other techniques when quantitative assessment of BMF is required in clinical and research settings. In current clinical work, the assessment of BMF by MRI is mainly based on subjective judgment, which is strongly influenced by subjective individual factors and has low sensitivity. Our study may have implications for changing this situation.

Several previous studies on vertebral BMF have found that BMF grows progressively higher at lower vertebral levels [19, 20]. The results of the present study were similar, indicating that this pattern applies consistently to different spinal types. We believe that this may be a general phenomenon that is not easily influenced by other factors, but the causality needs to be further investigated.

The present study also found that the BMF of the same level gradually decreased from type 1 to type 4, without exception, at all five lumbar levels. Previous studies on IVD and sagittal spine alignment found that types 1 and 2 were more prone to IVD degeneration than types 3 and 4 [21, 22]. The results of the present study also showed that types 1 and 2 had a higher BMF than types 3 and 4 at L4 and L5. We believe that the spine as a whole is more prone to IVD degeneration along with BMF deposition in the vertebral body in types 1 and 2 than in types 3 and 4. We suspect this is due to the greater physiological curvature of the lumbar spine in types 3 and 4, which is more conducive to pressure dispersion, resulting in lower vertical pressure on the vertebral body and disks and a lower incidence of spinal degeneration. Roussouly et al.[21] also stated that when the lumbar spine is hypolordotic and flat, the action of contact force is mainly on the anterior column (vertebral bodies and disks) and its distribution favors the resultant force perpendicular to the disks, increasing the disk pressure. On the other hand, if lumbar lordosis is hypercurved, contact force acts mainly on the posterior elements (facet joints and spinous processes) and increasing the stress on the facets and decreasing the vertebral body and disk pressure. However, the results showed no significant difference in FF across spinal types at L1-L3, which we speculate may be because the present collection was from an asymptomatic young and middle-aged population, in which the degree of spinal degeneration was likely to be mild. The findings may indicate that the progression of vertebral body marrow fat deposition begins in the lower lumbar spine.

Our study found that type 1 had significantly higher BMF at L4 and L5 than type 4. We presume this is due to type 1 has a smaller and lower lumbar anterior convexity arc whose apex is at the level of the central L5 vertebral body, such that the pressure on L4 and L5 is increased and presents a risk of vertebral fat deposition under the influence of various factors. Of course, this needs to be confirmed by further longitudinal studies in the future. Additionally, we speculate that it may be a factor in the increased incidence of IVD degeneration in type 1 at the L4/5 level. Ji et al.[23] found that the severity of IVD degeneration increases with the fat content of adjacent vertebrae, and this relationship is particularly pronounced at the L4/5 lumbar level. It has also been suggested that the apex of lumbar lordosis in type 1 is located at the level of the central L5 vertebral body and that there is increased stress in the small joints at sites with excessive lumbar lordosis, such as L4/5 and L5/S1. A recent study by Krug et al.[14] identified an increase in isthmus stress, especially in L5, which eventually led to L5 isthmus cracking and susceptibility to early degeneration of L4/5 and L5/S1. All of this is consistent with our findings.

In addition, previous studies have generally concluded that type 2, known as “flatback,” is the most susceptible to IVD degeneration, whereas our study showed that type 1 had the highest BMF [11, 21, 22, 24]. Spinal degeneration is a multifactorial disease that is closely related to age, body mass, muscle mass, intervertebral space height, bone mass, and biomechanics; thus, it is possible that the progression of vertebral body and IVD degeneration varies across spinal types and that the magnitude of each factor's effect varies. Further studies are required to explore whether there are differences in the correlation between vertebral body fat content and IVD degeneration in different spinal types.

As spinal degeneration is a major public health problem, several drug experiments and new technology studies related to this condition have been carried out in clinical settings. For example, a previous study by Luo et al.[25] found that alendronate can delay the progression of IVD degeneration by improving bone metabolism and vertebral osteoporosis, and another study by Liu et al.[26] found that fullerenol nanoparticles, as free radical scavengers, can prevent fatty bone marrow deposition and inflammatory responses in the vertebral body during IVD degeneration. Our study found that vertebral BMF was higher in types 1 and 2 than in types 3 and 4, and other studies of IVD degeneration have also found that the former are more prone to degeneration. Therefore, patients with types 1 and 2 could be monitored clinically for targeted BMF, and if high BMF is detected, appropriate prophylactic treatment may be available to prevent or slow the progression of degeneration. In this study, the BMF of individual vertebral levels of different spinal types was found to be somewhat different on quantitative MRI, and these changes generally emerged earlier than the morphological changes detected by conventional MRI sequences. With the development of medical technology, precision medicine is the direction of the future. In the future, it may be possible to precisely intervene at specific sites, for example, by injecting only the appropriate drugs into specific vertebrae, thus improving the microenvironment while reducing the side effects of systemic medication. This is of great significance for individualized treatment of different types of patients, and the results of this study have some reference value for this.

Our study had several limitations. First, our study was cross-sectional and sample was from a single center, a future multicenter longitudinal study investigating how sagittal spine alignment and vertebral BMF actually affect each other would be valuable. Second, our results may be limited because they were based only on the BMF, while perfusion factors associated with cellular and microvascular density were not evaluated; Karampinos et al.[2] concluded that these factors need to be considered in combination. Third, because there have been few studies on this topic, the study was conducted in asymptomatic young- and middle-aged volunteers to reduce confounding factors. Future studies including elderly individuals and patients with degenerative spinal diseases will be conducted. In addition, the exact model is unknown because the vendor does not disclose it.

## Conclusion

In conclusion, our findings revealed that vertebral BMF is associated with sagittal spine alignment, which may serve as a new additional explanation for the association of sagittal alignment with spinal degeneration. Our data may be helpful for increasing awareness of the relationship between spinal subtypes and vertebral BMF.

## Data Availability

The datasets used and/or analyzed during the current study are available from the corresponding author on reasonable request.

## Abbreviations

BMF: Bone marrow fat
MRI: Magnetic resonance imaging
FF: Fat fraction
IDEAL IQ: Iterative decomposition of water and fat with echo asymmetrical and least-squares estimation quantitation
ANOVA: Analysis of variance
LSD: Least significant difference
IVD: Intervertebral disk
PACS: Picture archiving and communication system
BMI: Body mass index
LL: Lumbar lordosis
TK: Thoracic kyphosis
ROI: Region of interest
SD: Standard deviation
